# Development of a Provisional Model to Improve Transitional Care for Female Adolescents with a Rare Genital Malformation as an Example for Orphan Diseases

**DOI:** 10.1155/2014/913842

**Published:** 2014-12-03

**Authors:** Elisabeth Simoes, Andrea Kronenthaler, Christine Emrich, Monika A. Rieger, Kristin Katharina Rall, Norbert Schäffeler, Hanna Hiltner, Esther Ueding, Sara Y. Brucker

**Affiliations:** ^1^Centre of Women's Health, University Hospital Tuebingen, 72076 Tuebingen, Germany; ^2^Women's Health Research Institute, 72076 Tuebingen, Germany; ^3^Social Medicine Staff Unit, University Hospital Tuebingen, 72076 Tuebingen, Germany; ^4^Institute of General Practice, University of Tuebingen, 72074 Tuebingen, Germany; ^5^Institute of Occupational and Social Medicine and Health Services Research, 72074 Tuebingen, Germany; ^6^Department of Psychosomatic Medicine and Psychotherapy, University Hospital Tuebingen, 72076 Tuebingen, Germany

## Abstract

Deficits of care exist during the transitional period, when young people with ongoing needs of support to achieve their physical, social, and psychological potential are entering adulthood. This study aims to develop a patient oriented, structured provisional model to improve transitional care for adolescents with *Mayer-Rokitansky-Kuester-Hauser-Syndrome* as an example for orphan diseases, where problems of access and continuity are even more complex. The study is funded by the German Federal Ministry of Education and Research (BMBF-Funding Code 01GY1125). The target patient group are young females with this disorder, treated at the Centre for Rare Genital Malformations in Women (ZSGF), University Hospital of Tuebingen. The study comprises five phases: an appraisal of literature, assessment of patients (*n* = 25), parents', partners', and health and social care providers' (*n* = 24) needs and experienced deficits in care and support in a qualitative approach, construction of a provisional model via scenario technique, followed by communicative validation (including interested public, *n* = 100), preference finding, and identification of patient-oriented quality aims for follow-up. Quantitative data from questionnaires and chart review (as sociodemographic data, nonresponder analysis, and preference rating) are worked up for descriptive statistics. The results provide a platform for the development of future multidisciplinary transitional intervention programs in orphan diseases.

## 1. Background

### 1.1. Health Care Issues

Transition in health care means the period from adolescence into adulthood, where people with special medical care needs move from child- to adult-centred care. Transition is a multidimensional, multidisciplinary, and active process that addresses the medical, psychosocial, and educational needs [1] of adolescents (defined by the World Health Organization as 10–19 years of age [2]). There are a growing and significant number of young people, who are entering adulthood with ongoing needs of support to achieve their physical, social, and psychological potential [3]. The German Advisory Council on the Assessment of Developments in the Health Care System reveals in its Special Report 2009 [4], based on international experience, local German projects and scientific literature, deficits of care during the transitional period, primarily caused by shortcomings in coordination. The available data do not allow reliable statements to be made on the extent of over-, under-, and misdirected provision of care and the special expectations of the patients, who may be in need of support alongside the medical treatment regarding other questions during puberty as especially sexuality, separation from the parental home, social integration, and career planning.

### 1.2. Rare Genital Malformations as Orphan Disease

Disorders in which transition is important include complex genital malformations in females. They belong to the so-called orphan diseases (less than 5 to 10 per 10000 people are affected). More than 7.000 of the 30.000 known diseases belong to the entity of* orphan diseases*. Orphan diseases all together affect many persons; about four millions of patients live in Germany [5]. For the affected patients and their families they often mean a considerable burden [6]. The incidence of the* Mayer-Rokitansky-Kuester-Hauser-Syndrome* (MRKHS), characterized by a congenital missing of uterus, cervix, and upper two thirds of the vagina, is estimated by 1 : 4.000–5.000 female life births [7] or 1 : 14.000 up to 1 : 50.000 of all women in the population. Genital malformations in females are diagnosed at different times between birth and adulthood, according to type of disorder, but very often too late. Diagnosis of MRKHS is still preceded of a tedious patient career, including wrong diagnostic and treatment attempts (40,9% [8]). Often diagnostic approaches coincide with the transitional period. Congenital genital disorders affect in an outstanding manner—besides (psycho-) social and personal coping—the sexual and personal development of the adolescent. In all these respects, MRKHS can be looked upon as a model for transition problems in orphan diseases. Transition into adult life as time of profound psychological and social change for all young people and their families is even more difficult for adolescents with long-term care needs and a rare disease. Transition is complicated by changes in needs and difficulties in access to proper medical and suitable (psycho-)social services. The known problems of the transitional period as discontinuity in medical care, insufficient accessibility, and missing social support for personal development may prove even more pronounced than for adolescents with common diseases. Central aspects of the adolescent phase,* sexuality*,* and construction of gender identity* are affected by the studied condition. In view of the generic nature of puberty and transitional care issues [9], the results concerning needs and experienced deficits—as far as these central topics are concerned—have relevance for young patients with other* childhood onset chronic illnesses*. Furthermore,* young females with genital mutilation *may benefit from the results of improved structures for and provision of care, support, and networks for diagnosis and treatment for females with genital disorders. Most of these young females will have a migration background. Their number is increasing. According to European Parliament estimates, 500 000 girls and women living in the European Union have been subjected to female genital mutilation (FGM), while a further 180 000 are at risk every year [10].

### 1.3. Current State of Research

Transitional programmes are in place in several foreign countries as in Australia, the United Kingdom, or the USA; in Germany there are only to some extent local solutions [4]. Some principles of transitional care are well consented. Transition needs to encompass inter- and intra-agency communication and coordination. Integrated primary care and social service involvement throughout the transition process is looked upon as an important aspect of transition [11]. Transitional care offers need to be age-adjusted. Research has provided special concepts for “youth specific healthcare,” in answer to a basic WHO conception [2]. A recent literature review highlighted eighth domains that stood out as central to young people's positive experience of care (accessibility of health care; staff attitude; communication; medical competency; guideline-driven care; age appropriate environments; youth involvement in health care; and health outcomes) [12]. Special importance is attributed to conceptual components preserving continuity of care [13]. However, there have been only limited evaluations of transitional care programmes that support beneficial outcomes in a number of chronic diseases, which affect more persons. Reviews of international transitional care practice continue to report a paucity of robust evidence. The conditions involved vary (e.g., cystic fibrosis, diabetes mellitus, and juvenile idiopathic arthritis), outcome measures varied accordingly. It is not clear how generalisable the successful studies, for example, regarding diabetes mellitus, will be to other conditions. Rare diseases were not considered so far [3, 14]. Many factors being influential in the transition are described in literature, as for example, disorder treatment, previous adherence and experience, the peer groups attitudes, psychological factors, social demographics, regional characteristics, current services organization, and experience of it or the relationship to parents or partner [3, 15]. A number of practice components which may promote continuity of care in general can be identified; however, supporting evidence for each type is variable. The various instruments to support coordinated transitional care (e.g., transitional consultation hours, adolescents' wards and training programmes, and self-help via websites and bulletin boards) developed abroad and designed for different conditions give valuable hints [16]. For instance, special consideration will deserve the six core elements of transition, set up in a quality improvement intervention modelled after the American Academy of Pediatrics/American Academy of Family Physicians/American College of Physicians Clinical Report on Transition [17]. Identifying the various practices and potential influential factors is of limited utility so far, as they are not tailored to the special needs and situation of the target patient group. Programme elements characterized by successful evaluation call for translation and adaptation to the framework of the national health care system prior to implementation. The beneficial experiences in other countries, pointing to the establishment of special provisional structures, should be taken into account. An interdisciplinary approach is mandatory [1], with involvement of all affected parties. Addressing experts only may fail to uncover important issues [18]. Hence, the model for transitional care needs to be developed in response to prior need assessment in different target groups.

### 1.4. Empowerment for Sustainable Self-Management

Besides the qualification and the experience of the medical professionals, patient's adherence is essential to the success of transition [19]. Often there are barriers (e.g., shame, deficits in information, and reliance) to effective communication. There is a broad consensus in literature that an additional process of patients' empowerment is needed: the young person in transition should be qualified through information to be actively involved in treatment, choice of measures, health behaviour, and health promotion, becoming an “expert on her own disorder.” Strengthening self-control and self-reliance as well as promoting communicative skills are reported to support continuity [1]. Some of these aspects, such as the provision of opportunities for adolescents to visit the clinic alone or to decide who should be present during consultations, were especially addressed in a Dutch study to evaluate a quality improvement program to improve transitional care [20]. The program resulted in improvements within a short time, thus outlining the chances of a patient-oriented tailoring of provisional offers and programs. The inclusion of patient-reported aspects and quality of life issues is central to present understanding of health care quality [21]. Integrating aspects of cultural diversity into empowerment concepts is expected to be another aspect supportive to adherence and continuity.

### 1.5. Special Issues

The situation of female adolescents with MRKHS as a rare disease is complicated by additional problems: knowledge of this condition tends to be low in public and even limited in health care providers. Genital malformations in females are superimposed by gender issues and imply tendencies towards stigmatisation and social isolation. Therefore, transitional needs are perceived wide-reaching and to necessitate a program of care that addresses all these special concerns. Transitional concepts developed for the context of other and more frequent medical conditions therefor may not or only in part be appropriate. 

*Puberty and Identity.* Besides the socialisation-process in the childhood and afterwards, sexual identity, as a part of the personality, is most notably constructed within the peer-group during puberty and needs being reconstructed permanently later on. This phase is difficult for all teenagers, since the social pressure to meet peer expectations is high. Puberty is a crucial period in the process of constructing the own identity as a young woman. For those who suffer from a physical disorder or mental disease, this period is even more difficult. During puberty, the girls affected by MRHKS are surrounded by peers for whom sexuality becomes an issue, sexual interests start growing, and first sexual experiences are made while they cannot keep up due to their physical condition [22]. This applies accordingly to first considerations about the own identity as a female, which may include the wish to have a child later on. These circumstances call for a targeted transitional care concept, turning to these special concerns to avoid developmental imbalances [23].

*Gender Aspects.* This study addresses young females. Extremely little is known about psychological sequelae of genital disorders in female patients. The choice of this rare disease allows highlighting the special needs for care and support of female adolescents during the transitional period and beyond. There may be special needs to empower these adolescents by facilitating independence and future self-management. Gender issues include societal expectations. The affected adolescents often encounter a lack of understanding and rejection within the context of their family, partnership, at school, or even finding themselves exposed to stigmatisation. Concepts have to face these challenges, too. 

*Avoiding Health Inequity.* Transition is complicated by further problem zones as socio-economic and regional factors, which increasingly enhance inequity in health chances four young persons, as the BELLA Study reveals for Germany [16]. Those factors interfere by reducing health chances via barriers to access specialist care and medical (university) centres [24], the last being essential for patients with orphan diseases. The experience of being lost in the system often coincides with lower health literacy. Informational needs may include health insurance issues. Socioeconomic and regional factors, superimposed by gender issues and attitudinal barriers, interfere by, for example, further reducing educational chances and limiting career prospects.

### 1.6. Centre for Rare Genital Malformations in Women (ZSGF)

To improve care provision for patients with rare diseases, the University of Tuebingen has inaugurated a Centre for Rare Diseases (ZSE) in January 2010. The ZSE includes a subcenter for congenital dysplastic disorders in females: the first and only German Centre for Rare Genital Malformations in Women (Zentrum für Seltene Genitale Fehlbildungen der Frau, ZSGF). The ZSGF is, as the University Women Hospital and the Women's Health Research Institute, integrated in the Centre for Women's Health of the University of Tuebingen. The ZSGF has a longstanding special experience in treating congenital genital malformations [25–28]. The treatment offers are supported by a multidisciplinary team with specialists of pathology, human genetics, psychosomatic medicine, endocrinology, and the paediatric department. Together they have been striving for continuous improvement of their offers of treatment and support for this special group of young females. Based on this outstanding experience, this study aims to add to the centre's structure a concept for integration and networking (e.g., with other health professionals as general practitioners), meeting the challenges of suitable targeting—avoiding inequity in health chances, providing accessibility of health care provision and tailored information in a changing society [29] to those who would not find their way readily to the needed specialist care.

### 1.7. Aims

Diagnosis, treatment, and research regarding orphan diseases need a close cooperation between specialists of different medical and social professions. Regional variation in health care facilities and socioeconomically based disadvantages call for tailored concepts, new provisional structures especially for young patients with rare diseases, here exemplified by persons affected by MRKHS. Considerable scope remains for

- tailoring of targeted offers for these patients during the transitional period,
- improvement in access, processes, cross boundary networking, and information to reduce shortcomings in coordination within the health care system and avoid inequity in health chances.

The principal research question of the study TransCareO (BMBF Funding-code 01GY1125; development of a provisional model to improve transitional care for female adolescents with genital malformations as an example for orphan diseases) is as follows: which targeted and coordinated provision of care, structural components, and support will be adequate to improve transitional care for the target patient group?

It includes the following questions.

- Which are the special needs of care and supportive measures of female adolescents with genital malformations in the transitional phase?
- Which deficits and barriers in provision of care, additional support, and communication are experienced by the patients/affected persons?
- Which deficits in care and support exist currently in the perception of providers, parents, and other interested parties, for example, patients' organizations?

Based on the study results to these questions, the needed and preferred improvements in health care, support, and information will be identified, with respect to the aims of good transitional care.* Quality aims* regarding transitional care in this orphan disease will be identified together with the patients/affected persons and persons engaged in care and support. The quality aims for transitional care, as summarized by the German Advisory Council on the Assessment of Developments in the Health Care System in their Special Report 2009, will be observed [4]. In addition, quality aims identified for other conditions are retrieved from literature and taken into account. Special attention will be given to core quality issues as for example, confidentiality, communication, information-giving, partnership, and respect, which are reported to rank high by young people [30, 31]. Patient oriented outcome measures will be identified for use during follow-up.

## 2. Design/Methods

### 2.1. Design of the Study

A mixed methods design is chosen to address the wide variety of aspects. Following an evaluation and appraisal of the literature, the qualitative approach deals with the experiences and attitudes of different target groups; quantitative elements are included for the estimation of impact. Due to the complexity, heterogeneity, individual-centred and evolving nature of transitional care provision and as patients' and expert insights, for example, experiences, behavioural aspects, emotional attitudes, and preferences are focused, they need being addressed by a qualitative approach. It allows the researchers to explore, for example, complex attitudes as the experience of disappointment, satisfaction, and quality. Personal expert interviews, guided by female researchers, seem appropriate for the targeted patient group, as the subjects may be highly individual. Parents, partners, and experts, who have been involved in care and support for the target patient group, may be encouraged by a guided telephone interview to contribute out of their experiences and knowledge. All interview partners receive a short questionnaire before the start of the interview. This questionnaire will collect basic data for example, regarding region of residence, medical discipline. Persons invited to take part in a telephone interview, but who are not ready to follow the invitation, are also asked to provide these basic data. The final workshop will be held in Open Space Technology [32], which seems appropriate to get insights in preferences, public views, practicability, and feasibility issues. In this context, the participants will be addressed by questionnaires designed for satisfaction and preferences finding.

### 2.2. Description of the Study Population

The different target groups and the intended samples are shown in Table 1. The* target patient group* represents female adolescents with MRKHS, a condition affecting sexual and social development in an outstanding way. As the study focuses on patients with a rare disease, absolute numbers tend to be small (hard-to-reach group). As the sample should provide maximum heterogeneity* in patient and parent characteristics, including social and educational background, patient career, experience with the disorder, family structure, national and cultural background, region of residence, and regional provisional structure*, up to 25 interviews may be necessary.

*Target expert group I* comprises persons involved in the process of care and support (medical professionals as general practitioners, paediatricians, gynaecologists, and other persons, as, e.g., parents, partners, social workers, self-help groups, and health insurances); eligible persons should possess personal experiences with the diagnosis and treatment of the disorder. Maximum heterogeneity in the sample (e.g., professions, regions, administrative areas, relation to the affected person, age, and gender) will be strived for.

Further groups (e.g., general practitioners, politicians, and teachers) are part of the enlarged* target expert group II* in Phase 5. They contribute public views, may be engaged in the process of implementation, and act as multiplicators.

### 2.3. Phases of the Study

**Figure 1:**
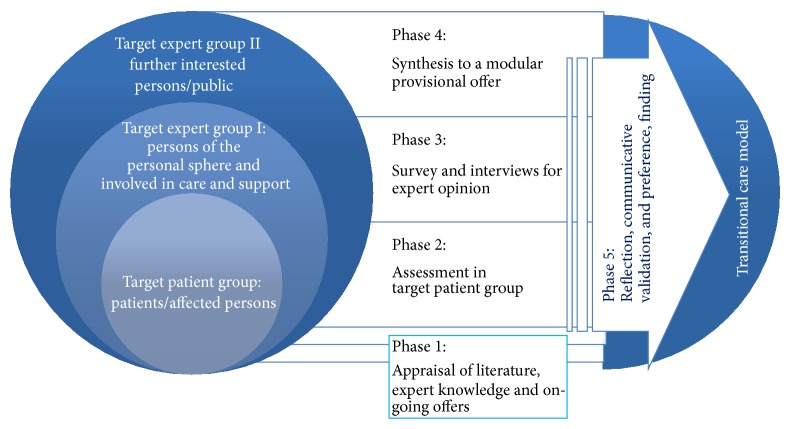
Phases of the study, overall view.

The study will consist of five phases: the first phase is dedicated to the search and appraisal of available knowledge and experience, the second assesses patients' and partners needs and experiences, and the third and fifth phases address persons involved in health care and (psycho-)social support for the target patient group and further interested parties. This triangulation will provide different perspectives to enhance validity. During the fourth phase, all results gathered will be used to construct scenarios for an improved provisional model (templates for centre and network), which, in the last phase, in a reflection loop, will be subject to final communicative validation (see below) and preference finding (Figure 1). 

*Phase 1* (Evaluation of literature, appraisal of personal experience, and ongoing offers at the ZSGF/Tuebingen, research guidelines). Findings of a systematic examination of the evidence regarding good practice models and components for transitional care will be gathered with special focus on genital malformations in females but also regarding other conditions. The objectives are to (i) identify the key components of good practice for promoting continuity at transition (e.g., continuity of information, cross boundary, and team continuity), (ii) identify elements relevant to the scope of the provisional model, for response to needs of the target patient group, and (iii) to inform the conception of the interview guides. Material will be also sought from experts in the field in Tuebingen (expert-interviews with persons of different professional background) and the great variety of ongoing offers at the ZSGF (e.g., peer involvement, provision of information, specific service provision, online forum, and homepage).

*Phase 2* (Assessment in the target patient group). This part will provide insight in the spectrum of deficits in and needs of health care and support during this special stage of personal development. The interviews will also contain an assessment of health care utilization. The patients will be asked to give information about the following data: (i) number of hospitalizations and hospital stays, (ii) number of contacts with GPs, specialists, and out-patient departments of hospitals, (iii) prescriptions of therapeutic measures, (iv) health care needs, and (v) utilization of additional services (e.g., psychosocial counselling).

The data collection from patients will be done in guided patient interviews by female researchers. The interview guideline is developed in a working group including a young patient representative, led by a senior methodologist, on the basis of ZSGF-expert interviews and literature survey and pretested in the context of the ZSGF. In the working group, different medical and social professions and different age groups are represented. Patients will be invited to the university or will be visited by the researchers at home, according to preference. Each interview will last about 90 minutes. Expert interviews with scope for narrative elements will be held (and audio-documented, according to consent). As part of the patient interview, a short questionnaire will be performed (administration in face-to-face interviews) to collect sociodemographic data at baseline including age, ethnicity, and socioeconomic status, oriented on the European socioeconomic classification (ESEC) (https://www.iser.essex.ac.uk/archives/esec/user-guide). 

*Phase 3* (Survey and interviews for expert opinion). Various stakeholders in the process of transition will be asked for their expert opinion: general practitioners, paediatricians, gynaecologists, parents, partners, social workers, self-help groups, women's organizations and health insurances (statutory and private) will be invited to take part. Organizational behaviour and cross boundary interaction will be of special interest. Regarding the subgroup of medical doctors as interviewees (treating patients in the out-patient setting), the interviewers will also collect the following data about the attending specialist: (i) medical discipline, (ii) location of clinic, (iii) age, (iv) gender, and (v) professional experience.

The data collection from these experts involved in care and support of the target patient group is done via a short questionnaire (sent per mail) for self-completion and additional invitation for guided telephone expert interviews. To gain a broad spectrum of aspects, the intention is to include 24 participants, (12 medical professionals and 12 other experts). Each interview will last up to 30 minutes. In the interview guide for the telephone interview also aspects gained out of the results in the patients interviews in Phase 2 will be included. Group-specific guidelines (parents, partners, and medical and other experts) are developed in working groups guided by a senior methodologist. In these working groups different medical and social professions and different age groups are represented. Group-specific pretests are done.

The following topics will be approached in the interviews of Phases 2 and 3.

- Which targeted and coordinated provision of care and support can be expected to improve medical, social, and psychosocial development of the adolescents?
- Which barriers are experienced in communication and information? What impact do they have on the time from onset of symptoms to diagnosis and final treatment?
- Which organizational components for structure and process of care in managing the transition, developed in response to a prior needs assessment in the target patient group, are expected to yield a positive impact?
- Which role should paediatricians, general practitioners, gynaecologists, parents, teachers, school psychologists, and women organisations have in the provision of care? Are they prepared? How do they have to be prepared?
- Which quality aims can be identified, which will foster quality assurance and improvement during further evaluation and implementation?

It is planned to perform one or, if necessary (e.g., due to insufficient heterogeneity), two waves of data collection by patient and expert interviews.

*Phase 4* (Synthesis to a modular provisional offer). In the previous phases (1–3) identified components of favourable practice will be synthesized to models as scenarios (based on Geschka et al. [33, 34]). The scenarios will contain different modules. The modules apprehend the main categories disclosed as deficiencies or necessities. Interventional modules may address (1) structural development, (2) training and skill development, (3) health education, (4) information for persons involved in medical care and support, for example, practitioners and teachers, (5) guidance of parents, (6) offers for partners, (7) meeting and exchange with peers. The modules comprise elements suitable for a transitional template for the ZSGF and modules addressing provision of group- and age-specific information and networking of professionals for medical and social support. 

*Phase 5* (Reflection, Communicative Validation, and Preference Finding). Communicative (or dialogical) validation, as a methodological step, involves sharing the researchers' understanding and interpretation of the data with the participants/interviewees to make sure that they agree with the researchers' interpretation and analysis of what they have said. Communicative validation adds value to the rigour of interview data analysis and also allows for further research insights.

In a workshop setting (Open Space Technology [32]), three to four provisional models are proposed for appraisal to patients, experts, and interested persons. All participants of Phases 2 and 3 are invited and, additionally, volunteers (interested persons, Target expert group II, Table 1). This final reflection loop aims, besides being part of the qualitative assurance system (see below), to a very close tailoring of the model in accordance with availability and improvement of the facilities and offers of the actual provisional German system. In Phase 5 data are collected via reports from topic-specific workshop-sessions and questionnaires within this context.

The scenarios comprise several elements of care and policy approaches. Satisfaction with the elements and preferences are measured. Participants in the workshop are asked to rate the extent to which each item represents best practice or current care using a 7-point Likert scale anchored by “strongly disagree” at 1 and “strongly agree” at 7. Anticipated satisfaction with each item is conceptualized as the difference between their “best” and “current” score. The ratings during the communicative validation express expectations and preferences regarding “best care.” The difference between the ratings will inform about predilections and importance of elements, as expressed by the different groups (patients, parents, medical experts, and further interested persons). Thus, different points of view will be included: for example, the perspective of healthcare professionals and policymakers may contribute aspects of practicability and fit to the framework of the healthcare system. The results will be used for a final tuning of the model. After implementation later on, the rating may be repeated to inform about satisfaction with care according to the model. To demonstrate the impact of a coordinated transitional care programme for adolescents with juvenile idiopathic arthritis, “gap scores” were collected in intervals during implementation. In that study, the scores proved as a reliable tool to inform about progress in satisfaction [35, 36].

### 2.4. Recruitment Procedure

By 150 eligible patients out of the ZSGF patient documentation system are contacted (in written form, by mail and as applicable by e-mail) and asked to participate in the study. The centre guarantees the feasibility of the study, as there is only a prospective recruitment out of a considerable number of patients with genital malformations that can be approached, within different ranges of age, social background, and patient career, coming from all over Germany and beyond. All contacted patients who are willing to participate are included consecutively in the study, up to a number of 24. The criteria for selecting the study sample are primarily purposive regarding the diversity of opinions (see below), in case of low response according to feasibility as dealing with a hard-to-reach group. Due to the investigation of multiple factors, careers, and outcomes and the observational character of the study, there is no issue of statistical power to be considered. We expect due to experience with previous studies a response rate by 20% for the target patient group and a drop-out rate of 10% from baseline to last phase (workshop). The study centre began to recruit in February 2013.

Strategies that have been shown to enhance response rates (e.g., including stamped addressed envelopes for response, offers to communicate online for further details) are used. In case of interest, the patient will respond in written form to the ZSGF and receive written information, covering aims and procedures as, for example, data collection, processing, and storage as well as possibilities for cancellation. Patients are informed, that neither participation in the study nor non-attendance influnce their medical treatment or support and that providers taking part in actual medical treatment are not involved in data collection and analysis. In case of acceptance, participants will have to sign an informed consent form to participate in the study.

A strictly patient-centred recruitment is chosen: potential participants belonging to target expert group I are named by participating patients, as to ensure proximity to the target patient group. Patients hand out information about the study to these persons of their personal sphere or ask the centre to send information directly (e.g., to medical experts). Written consent of the patient/affected person is a precondition for all steps of recruitment, which are performed by the study centre with regard to target expert group I.

Participants of the communicative validation workshop in Phase 5 belonging to the target expert group II need not have been necessarily involved in treatment of support of the target patient group. These persons will be reached by public invitation to the workshop at the University Hospital. As experience shows, up to 100 participants can be expected.

### 2.5. Data Analysis

#### 2.5.1. Documentation of Morbidity

The morbidity of the patients will be registered as part of the patient interview and via chart review for nonresponders (see below).

#### 2.5.2. Qualitative Analysis

The audio-documented interview material is transcribed (rules according to Kuckartz [37]). Analysis will be processed using the software MaxQda. According to qualitative content analysis [38] key issues will be identified, labelled as codes, and sorted into categories. This approach allows inductive category development. Analysis will be done by two independent trained researcher teams; hereby, transcripts will be reviewed independently to confirm codes and categories. Disagreements are discussed within the researcher team until consensus will be achieved. The researchers do not occupy dual roles (clinician and researcher).

#### 2.5.3. Cross-Sectional Analysis of Quantitative Data

Recruitment and sociodemographic data, satisfaction, and preference measures will be used to develop descriptive statistical analysis. The data out of the questionnaires of Phases 3 and 5 will be worked up using SPSS/PASW.

#### 2.5.4. Variables under Study

The variables under study belong to the following groups: morbidity and sociodemographic data. The domain of morbidity includes type of diagnosis and medical care according to the diagnosis. Sociodemographic variables include age, gender, migrant status, marital status, education, and occupation and are collected to describe the groups involved in the research process.

#### 2.5.5. Nonresponder and Drop-Out Analysis

For all eligible patients in the ZSGF patient documentation system, who did not respond to the invitation to participate in the study, information will be retrieved by* chart review according to a collection sheet.* Age, morbidity, and a variety of socioeconomic data will be used to estimate the actual selection bias. Recourse and number of excluded patients per exclusion criteria will be documented. In case of drop out, time and (if available) the reasons why participation was not continued, are registered (e.g., cancellation of participation in study, relocation, etc.).

### 2.6. Quality Assurance and Safety

The study is conducted in compliance with the Helsinki Declaration. The study protocol was approved by the Ethics Committee of the University Hospital of Tuebingen in February 2013 (25.02.2013, Project number 422/2012B01). Procedures for prevention of insufficient data quality, detection of inaccurate or incomplete data, and actions to improve quality will be performed, for example, user reliability trainings, automatic plausibility, and integrity checks.

To enhance credibility, adherence to systematic analytical procedures is ensured for all processes of transcription, data collection, and analysis, including literature search and choice of quotes. Interviewer corroboration and peer debriefing is performed for qualitative research elements. Triangulation assures to gain different perspectives with different methods (qualitative and quantitative) on the studied provisional processes. A scientific advisory board supports the research process. Communicative validation aims to ensure the involvement of the affected groups in the research and validation process as well as the reflection by experts of different professions, enhancing the fit to the framework of the social, educational, and social security system.

Data security rules of the University of Tuebingen are followed. Participants anonymity and confidentially are ensured. Informed consent is sought (see above). Often the studied rare genital malformations are diagnosed during puberty (one of the leading symptoms is primary amenorrhea). Thus, part of the affected persons will be of minor age. In this case, the permission of the parents will be secured in addition to the declaration of consent of the interviewee.

Monitoring for the impact of the study on patients will be performed by the interviewers and counselling offered and given in case participants experience any discomfort or harm, though it is not expected that the study will expose the patients or other participants to any risk.

## 3. Discussion

This study is the first considering transitional care for patients with MRKHS as a rare congenital malformation and thus contributes to a previously scarce evidence base. The value of this research also extends beyond the MRKHS, in that many issues identified as important are generic to other childhood-onset orphan diseases and further genital disorders.

Turning to an orphan disease does not allow addressing large numbers; however, it meets with specific challenges. Society and the health care system as a whole have only recently become more alert towards orphan diseases, supported by activities of patients' organizations and also initiatives of the BMBF. Publications addressing orphan diseases have rapidly increased. About 80% of the rare illnesses are of genetical origin and become symptomatic at birth or during childhood. Most of them are chronic diseases, including handicaps and mean ongoing medical needs and social support [39, 40]. Patients with orphan diseases meet with an accumulation of problems, when seeking treatment and support. The studied disorder fulfills a number of criteria which makes it useful to some respect as* tracer condition*: it is clear to define, it is amendable to improvement of the condition by correct diagnosis and treatment, there are criteria for distinguishing between high and low quality care and nonmedical and medical factors could be identified. After evaluation the structured concept could be used for further launch and to establish similar offers at dedicated centres for this or other orphan illnesses, with adaptions also for other childhood-onset chronic illnesses. Structural improvements may also facilitate access to suitable medical care and social support for the increasing number of young females with genital mutilation, their problems being in a number of respects similar to those of young females with genital malformations (e.g., affection of sexual and social development, access to highly specialized gynaecologic treatment).

In 2009, around 9300 women with a congenital genital disorder lived in Germany [8]. Per year, about 70–80 girls are affected. The probability of a gynaecologist to meet a patient with this disorder is estimated by 10%, even lower for a general practitioner. In contrast, 240 highly specialized interventions have taken place in the Department of Gynaecology at the University of Tuebingen since 1999 [41], offering a long- and outstanding experience. It needs being made use of for other patients seeking treatment—by structured information—to providers and public about the special facilities and offers and by establishing networks of medical and social professionals (including, e.g., general practitioners as the gate keepers, social workers, and teachers) to guide the young patient in her search for help. Within a period of 12 months, a total of 43 175 users were counted on the ZSGF homepage [11]. This shows that internet based information plays a significant role for young people and informational need are high. Tailoring such internet offers according to needs would not only improve the accessibility of reliable information about this rare disease MRKHS, but could also increase the probability that fast proper medical treatment is chosen and applied.

This study is the first to provide* patient-reported data *in how far specific* provisional* measures can be expected to meet MRKHS adolescents' needs with regard to good health care and supportive offers during the transitional phase. No previous study integrated the* various perspectives* of patients, parents, partners, and professionals engaged in care and support concerning the improvement of transitional care in young females with MRKHS or other orphan diseases internationally or within the framework of the German health care system. The diversity of offers for transitional care developed with respect to other more frequent medical conditions and the uncertainty concerning the special needs of support did not allow research-based recommendations regarding good transitional care in orphan diseases so far.

### 3.1. Expected Results, Use, and Implementation

The results will allow the design of structures and components of a provisional model tailored to the transitional situation of adolescents with MRKHS. The German National S1 Guideline “Weibliche Genitale Fehlbildungen” (female genital malformations) of the German Society of Obstetrics and Gynaecology (Deutsche Gesellschaft für Gynäkologie und Geburtshilfe, DGGG) does not consider provisional structures. The results may add to the* prospective S2-Guideline* some regards contributing to the improvement of access, alertness, cross boundary networking and information standards. Aspects of support regarding* sexual and social development in the transitional phase* of female adolescents are focused, where there is an additional considerable lack of knowledge.* Quality aims* will be identified. Patient-reported aspects will play a major role. The identification of aims together with patients/affected persons in a* participatory* process can be expected to promote patient-centred quality assurance during a prospective phase of implementation and evaluation.

For the first time, partners in addition to parents will be included in the process to express their special needs and to identify from their perspectives quality aims for health care and support during the transitional phase of the target patient group. Concerning those components of care and support, rated as essential for “best practice” in the communicative validation process and insufficiently realized so far, channels for implementation will be sought. The expected variety of organizational components for structure and process of care in managing transition may call for implementation through several additional channels beyond regular scientific publication (Table 2). Key components of the proposed provisional model may include age and developmentally appropriate informational material for adolescents, informational material for parents, medical and social staff, a departmental transition policy template for the subcenter, after evaluation apt for adaption by other centres (generalisation) as well as a template for networks.

### 3.2. Relevance and Impact on Health Care

While long and difficult patient careers may in part be attributable to the general paucity of services in these special fields, it also reflects a failure to manage and integrate care effectively so that a significant proportion of young people with ongoing needs become dislocated from the care system during the transitional period [42]; the negative impact for the individual patient and the health care system, by facing costs due to the sequelae, are at hand. So, results are of interest for the health care system and its quality of transitional care. A concurrent reduction of expenses may be expected, by avoiding delayed or wrong diagnosis and treatment and by preventing sequelae regarding physical and mental health through unfavourable patient careers (including the individual pain, loss, and public costs). Health insurances may derive concepts for case management, to improve care along with approaches to cost reduction. Organizational solutions found for one condition may be useful for other rare conditions, too, thus increasing the impact.

## 4. Conclusions

Internationally, a considerable knowledge gap exists regarding the transition period of adolescents with MRKHS and other orphan diseases and evidence for provisional approaches supporting beneficial outcome.

TransCareO will be the first study to provide a comprehensive assessment of the provisional situation and in addition a full examination of the sociocultural findings, values, beliefs and attitudes, and competing demands that influence adolescents with MRKHS (as an orphan disease) in transition, characterizing the baseline data for an implementation and evaluation study [43]. A model for transitional care will be developed, tailored to the identified deficits and needs, and prepared for implementation and evaluation at a centre, that is, the implementation of the model as transitional program. Thus, the project TransCareO comprises the first phases of a (complex) intervention to improve health care for female patients with rare genital malformations as an example for orphan diseases.

## Figures and Tables

**Table 1:** Study population and data collection, overview.

Target population	**Target patient group**: adolescents (according to WHO-definition) with the congenital malformation MRKHS *Inclusion criteria*: females with treatment for this reason at the German Center for Rare Genital Malformations in Women (ZSGF) during the past 10 years *Disease list for inclusion* (ICD 10 codes and description): Q 52 Other congenital malformations of female genitalia: *MRKHS-Q52.8 Other specified congenital malformations of female genitalia* *Exclusion criteria*: treatment for another gynaecologic disorder **Target expert group I**: persons at present or previously involved in health care and support of the target patient group: for example, partners, parents, medical professionals, and allied health professionals *Inclusion criteria*: involvement in treatment or (psycho-)social support of the target patient groups, within a period of the last ten years *Exclusion criteria*: no involvement in treatment or support **Target expert group II**: interested persons, for example, allied health professionals, social workers, teachers, health policy makers, statutory health insurances, The (German) Federal Joint Committee (Gemeinsamer Bundesausschuss), health administration professionals, not necessarily at present or previously involved in care or support of the target patient group *Inclusion criteria*: volunteer to take part in the public workshop For all participants the following *exclusion criteria* are applicable, in addition to the mentioned target group specific criteria: insufficient ability to speak and read German, insufficient ability to consent (e.g., debility), informed consent not given in written form

Sample size	Target patient group (Part 2): 25 Target expert group I (Part 3): 24 (12 medical professionals and 12 others) Target expert group II for communicative validation, preference finding (Workshop, Phase 5): up to 50 volunteers (in addition to volunteers out of the target patient group and expert groups I)

Data collection	**Phase 1**: evaluation of literature, expert-interviews with experts in the field and appraisal of on-going offers (e.g., at the ZSGF) **Phase 2**: guided patient interviews with narrative elements (audio-documented, interview by female researchers and health and social scientists), guided interviews with narrative elements with partners (interview by male researcher, psychologist, and health scientists) **Phase 3**: target expert group I addressed by a short questionnaire (per mail) for self-completion, additionally semi-structured telephone expert interviews **Phase 4**: scenario technique (Geschka et al. 1982 [34]) **Phase 5**: target patient group, target expert groups I and II included in workshop for communicative validation (Open Space Technology), addressed by questionnaires for rating and preference finding within this context

**Table 2:** Channels planned for utilization and implementation of the prospective results.

Setting	Prospective measure
ZSGF	Implementation and evaluation of the provisional template at and around the ZSGF

Other centres	After evaluation further launch of the transitional care program to other centres

Networking I	Developing professional knowledge and skill by information/teaching material, regional workshops for physicians, for example, gynaecologists, general practitioners, and paediatricians as well as other medical professions and allied health professionals

Networking II	Developing professional knowledge and skill by information/teaching material for professionals of educational and social professions (e.g., teachers, social workers)

Tailored information for distribution	Age-adjusted information material for young people for use for example, in schools Introduction of the topic in school books, for example, explaining genital malformations Tailored partner-and parent-information, for example, age-adjusted flyers Information for public and health policy makers

Internet platform	Youth-friendly internet offer Links to the centre's internet platform from professional societies and patients' and women's organizations
